# The Relationship between Species Richness and Evenness in Plant Communities along a Successional Gradient: A Study from Sub-Alpine Meadows of the Eastern Qinghai-Tibetan Plateau, China

**DOI:** 10.1371/journal.pone.0049024

**Published:** 2012-11-09

**Authors:** Hui Zhang, Robert John, Zechen Peng, Jianli Yuan, Chengjin Chu, Guozhen Du, Shurong Zhou

**Affiliations:** 1 State Key Laboratory of Grassland Farming Systems, School of Life Sciences, Lanzhou University, Lanzhou, Gansu, People’s Republic of China; 2 Department of Biological Sciences, Indian Institute of Science Education and Research, Kolkata, Mohanpur Campus, West Bengal, India; 3 Ministry of Education Key Laboratory for Biodiversity Science and Ecological Engineering, School of life Sciences, Fudan University, Shanghai, China; University of Alberta, Canada

## Abstract

The relationship between species richness and evenness across communities remains an unsettled issue in ecology from both theoretical and empirical perspectives. As a result, we do not know the mechanisms that could generate a relationship between species richness and evenness, and how this responds to spatial scale. Here we examine the relationship between species richness(S) and evenness (Pielou’s J′ evenness) using a chronosequence of successional sub-alpine meadow communities in the eastern Qinghai-Tibetan Plateau. These meadows range from natural community (never farmed), to those that have been protected from agricultural exploitation for periods ranging from 1 to 10 years. A total of 30 sampling quadrats with size of 0.5 m×0.5 m were laid out along two transects at each meadow. Using correlation analyses we found a consistent negative correlation between S and J′ in these communities along the successional gradient at the sampling scale of 0.5 m×0.5 m. We also explored the relationship between S and J′ at different sampling scales (from 0.5 m×0.5 m to10 m×10 m) using properly measured ramet-mapped data of a10 m×10 m quadrat in the natural community. We found that S was negatively corrected with J′ at the scales of 0.5 m×0.5 m to 2 m×2 m, but such a relationships disappeared at relative larger scales (≥2 m×4 m). When fitting different species abundance models combined with trait-specific methods, we found that niche preemption may be the determining mechanism of species evenness along the succession gradient. Considering all results together, we can conclude that such niche differentiation and spatial scale effects may help to explain the maintenance of high species richness in sub-alpine meadow communities.

## Introduction

Diversity is one of the most important community attributes which can determine stability, productivity and migration [1]. Diversity mainly includes two different aspects: species richness and evenness. Species richness, or the number of species, is the simplest measure of diversity and does not consider differences in species relative abundance. Species evenness or the similarity in species relative abundance in a community captures another aspect of diversity by determining diversity as a standardized index of relative species abundance [2]. The majority of studies exploring the causes and consequences of biodiversity have used species richness to represent diversity on account of its apparent simplicity compared to species evenness. However many investigations also indicate that species richness is a common cause of variation in relative abundance and diversity [3]. Furthermore the relationship between species richness and evenness can vary with change in key ecological processes such as competition, predation, and succession, each of which can alter proportional diversity through changes in evenness without any change in species composition [4], [5]. Hence some authors suggest that we should treat the two main components of diversity separately in investigating the determinants of diversity [3], [6]. Therefore, understanding the patterns and the mechanisms that determine the relationship between species richness and evenness can provide important information on biodiversity maintenance [7]. However the relationship between species richness and evenness across local communities remains a controversial issue in ecology because of the wide variation in both of empirical and theoretical patterns that have been reported [8].

Several theoretical studies have assumed that species richness and evenness should be independent or positively correlated. These studies date back to DeBenedictis [9] who argued that mathematical relationships between measures of diversity such as species richness and evenness constrain the correlations among these measures and that they should therefore be strongly and positively correlated. Similar predictions of strong and positive relationships among measures of community diversity have been made by other theoretical studies [1], [10], [11]. More recent investigations by Jost [12] suggest what DeBenedictis [9] had earlier pointed out – that species richness and species evenness are not independent components of community diversity and that the correlation is constrained by the underlying mathematical relationships. Jost [12] then proceeds to argue that we should consider a relative measure of evenness (relative to the maximum possible value for a given species richness) in comparative analysis of communities. Nevertheless, many studies have assumed that species richness and evenness are two independent indices [13], [14], [15], [16].

Empirical studies of the relationship between species richness and evenness have also reported contrasting findings. Earlier observational research suggested that species richness was positively correlated with evenness [10]. More recently other empirical studies indicate that species richness and evenness are strongly negatively [1] or independently [17] associated in plant communities. These contrasting theoretical arguments and empirical findings cannot be easily reconciled. We there need more research on the relationship between richness and evenness along different geographical gradients and in different communities across the world. Here we propose to investigate the relationship between richness and evenness in species-rich sub-alpine meadow communities in the Qinghai Tibetan plateau.

Species-area relationships have clearly demonstrated the effect of spatial scale on species richness [18], [19]. Similarly, many studies have investigated the influence of spatial scale on evenness [20], [21], [22]. Since both species richness and evenness depend on sampling area, the relationship between species richness and evenness may vary with spatial scale. Moreover, to our knowledge, the influence of spatial scale on the relationship between richness and evenness has not been investigated in detail. Here we also propose to explore the effect of spatial scale on the relationship between richness and evenness by employing sampling quadrats at multiple spatial scales (0.25 m^2^ to 100 m^2^ ).

Many investigations indicate that different abundance models combined with trait-specific methods can provide us a very powerful tool to explore the mechanisms of community assembly [23], [24], [25], [26]. If species richness and evenness of a community are related through fundamental ecological processes, then understanding the relationship between these two measures would shed light on the mechanisms that determine community structure. The trait-specific method focuses on specific functional traits [27], [28] to model the relationship between these traits and environmental gradients (e.g., [29]). Currently different studies have used different functional traits to test this method and to explore different ecological properties of communities [27], [28], [29], [30]. One important functional trait, the specific leaf area (SLA), reduces a fundamental axis of differentiation among species [31] from more acquisitive (higher SLA, higher obtain of resources) to more conservative (lower SLA, retain of resources within protected tissues) strategies. Furthermore, many studies have shown that SLA is highly correlated with local abundance and environmental conditions [32], [33], [34], [35]. Hence, we aim to explore the relationship between SLA and species relative abundance in various environments produced along the successional gradient.

Our study system, the sub-alpine plant community in the Qinghai-Tibetan Plateau, is particularly well suited to address important questions concerning species richness and evenness of communities. The communities are of high diversity, and investigations of community attributes are relatively easy [36]. Moreover meadow degradation is a serious threat to the biodiversity of the area, mainly due to agricultural exploitation and over-grazing in recent years [37], [38]. Hence, exploring the relationship between richness and evenness and their underlying mechanisms along the successional gradient can thereby provide better understanding and possible rules for restoration of this particular ecosystem. Therefore our specific objectives are: (1) to test the relationship between species richness and evenness empirically; (2) to explore the effect of varying sampling scale on the relationship between species richness and evenness; and (3) to investigate the mechanisms of community assembly in these successional meadow communities.

## Results

### The Relationships between Empirical Species Richness (S) and Evenness (J′)

**Figure 1:**
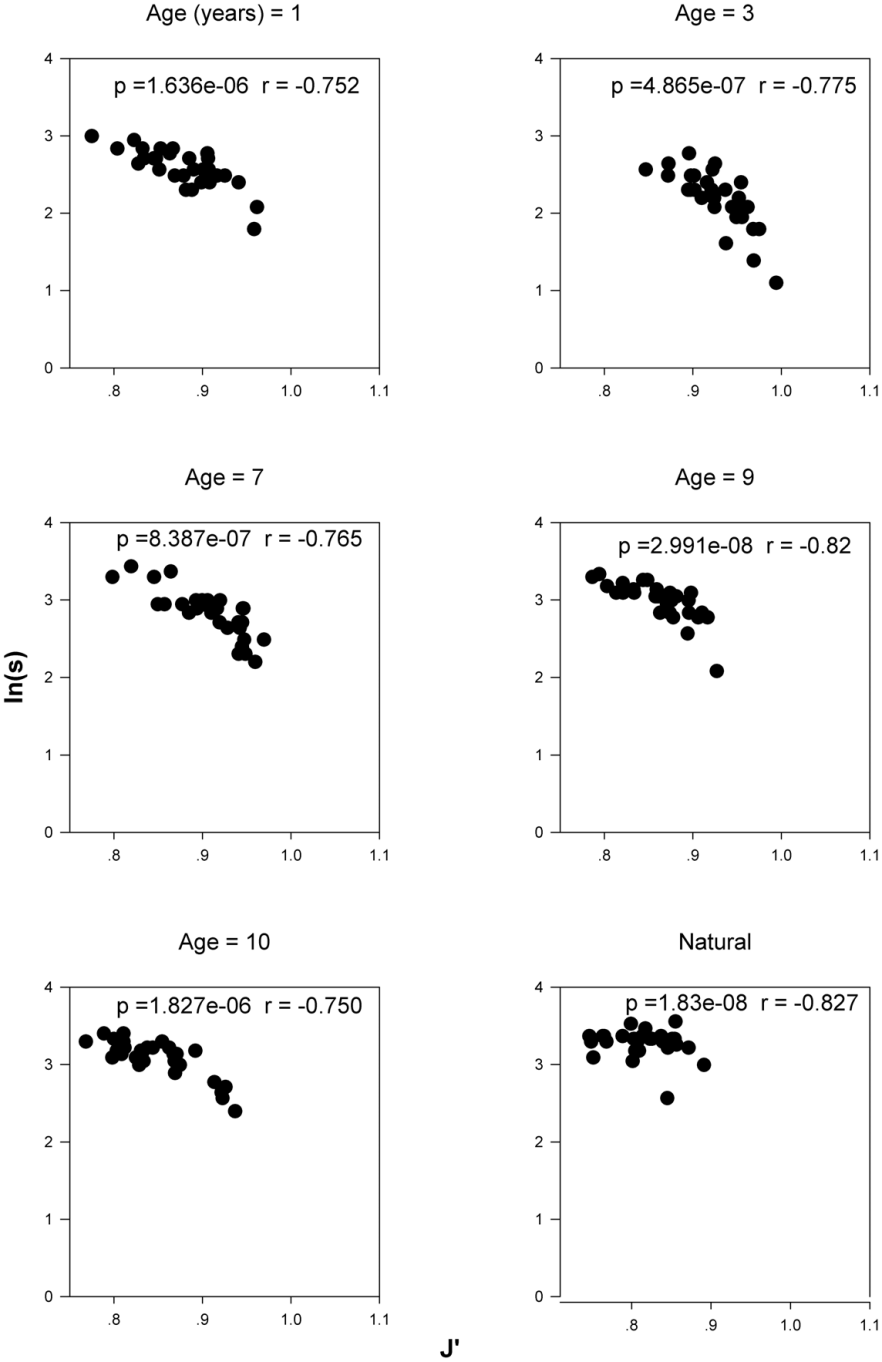
The relationship between empirical species richness and evenness with successional age. p, p-value; r, correlation coefficient.

**Figure 2:**
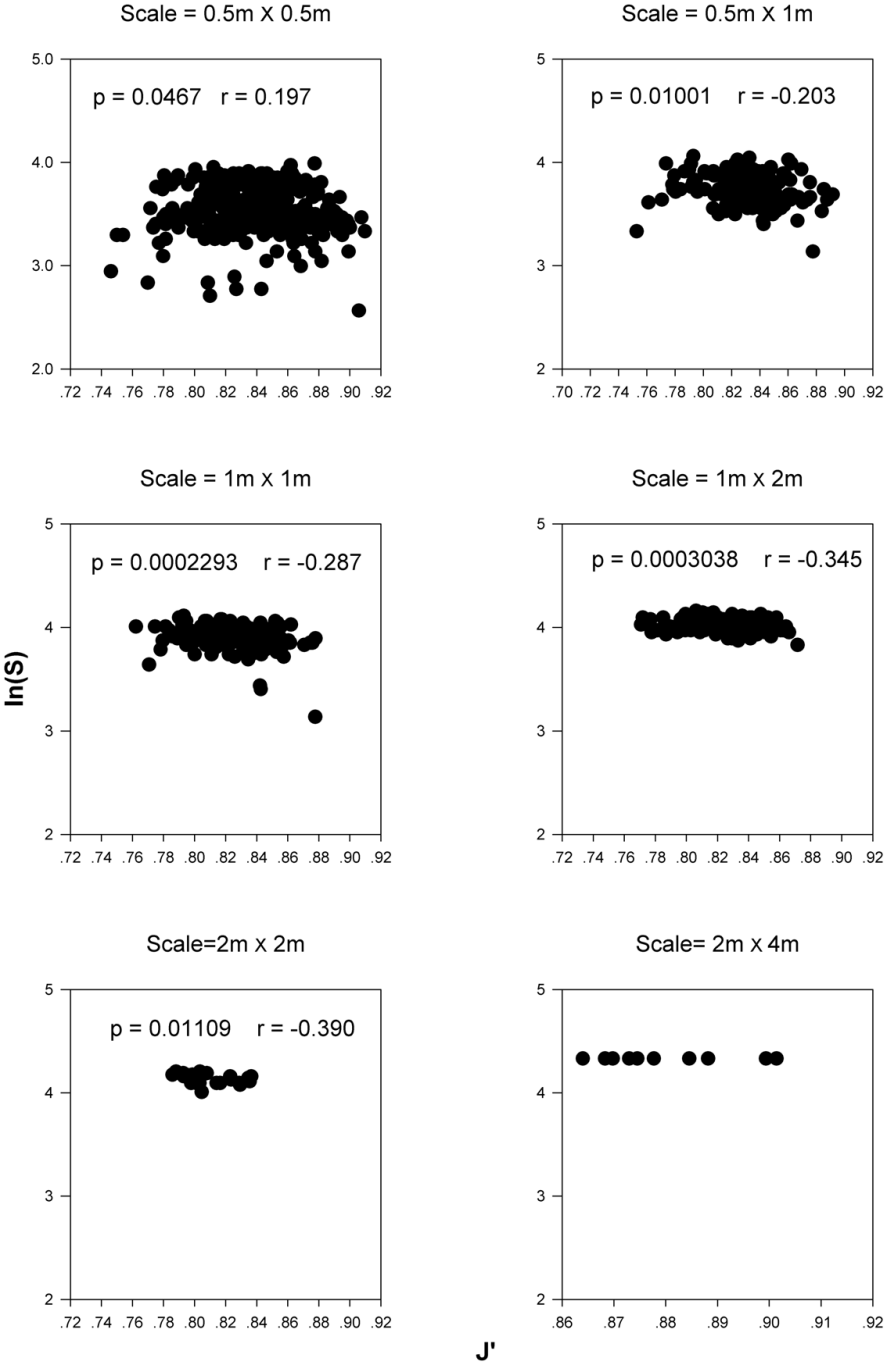
The relationship between empirical species richness and evenness at different sampling spatial scales. p, p-value; r, correlation coefficient.

Although evenness (J′) varies substantially along the succession gradient (P value of one-way ANOVA = 0.006, Table S1), all meadows show consistent and significantly negative relationship between S and J′ at the scale of 0.5 m×0.5 m (Fig. 1). When taking spatial scale into account, in the natural community, these negative correlations between S and J′ are maintained at small to medium spatial scales (≤2 m×2 m). However, S does not vary with J′ at scales larger than 2 m×4 m (Fig. 2).

### The Result of Model Fitting of the Observed J′

**Figure 3:**
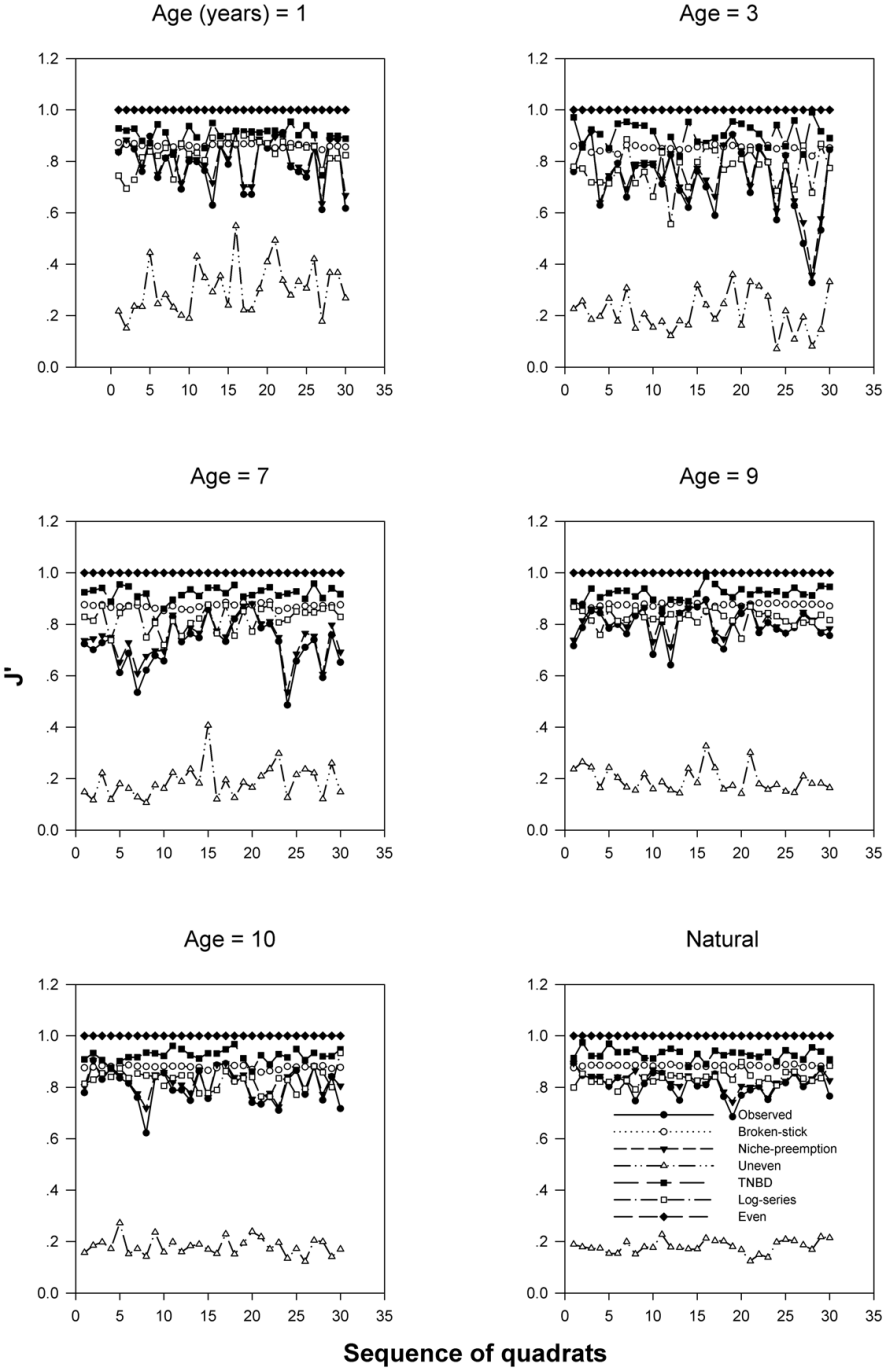
The value of the observed and respective fitted J′ of the responding 5 species abundance models of each of the 30 quadrats in the plot of the respective successional age.

Although there are significant differences in the observed J′ along the succession gradient, only the niche-preemption model can fit the observed J′s in these successional meadows at the scale of 0.5 m_×_0.5 m (Fig. 3 and Table 1).

**Table 1:** The p-value of the pair-wised bootstrap Kolmogorov-Smirnov test of the observed J′ and the five fitting ones predicted by the six responding models in meadows with different successional age.

	age = 1	age = 3	age = 7	age = 9	age = 10	natural
Uneven	1.87E-13	1.11E-15	1.87E-13	1.87E-13	<2.20e-16	6.88E-14
TNBD	3.24E-06	6.53E-09	3.00E-14	3.00E-14	8.25E-12	4.93E-13
Log-series	0.05	0.02	2.37E-05	0.01	0.05	0.02
Niche-preemption	**0.95**	**0.96**	**0.39**	**0.59**	**0.59**	**0.25**
Broken-stick	3.24E-06	1.97E-07	5.59E-11	3.27E-10	4.40E-08	3.30E-12
Even	1.87E-13	1.87E-13	1.87E-13	1.87E-13	1.87E-13	6.88E-14

### Changes in Species Traits along the Successional Gradient

Specific leaf area (SLA) varies significantly with successional age. In the initial stage of succession (one-year old meadow), SLAs were significantly negatively correlated with species relative abundance at the scale of 0.5 m×0.5 m, while this relationship shifted to positive in the later stages of succession (the natural communities and the meadow10 years after abandonment) (Table 2).

**Table 2:** The Spearman correlation coefficients (r) and p-value (p) of the Spearman correlation analysis of the relationship between SLA and species relative abundance along the succession gradient.

age = 1		age = 3		age = 7		age = 9		age = 10		natural	
r	p	r	p	r	p	r	p	r	p	r	p
**−0.66**	**0.043**	**−**0.02	0.94	0.01	0.97	0.19	0.43	**0.60**	**0.01**	**0.49**	**0.01**

**Table 3:** the species-abundance models which have different evenness in abundance.

Species-abundance model	specification
Most even	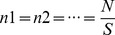
Broken-stick	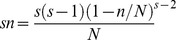
Niche-preemption	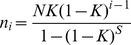
Log series	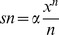
TNBD	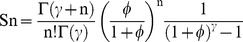
Most uneven	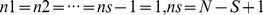

## Discussion

### The Relationship between Species Richness (S) and Evenness (J′)

In our study, consistent significantly negative relationships were observed between species richness and species evenness along the successional gradient. This is consistent with [17] which assumes that species evenness and richness in a grassland community were negatively related. Our results also support many investigations which argue that species richness can be determined by species evenness in abundance [7], [39], [40], [41]. Therefore, investigating the mechanisms that influence species evenness can also help us understand maintenance of diversity [7]. Stirling and Wilsey [1] concluded that Log(S) and J′ are always negatively related and that empirical observations could not be explained without including indirect effects. Moreover succession itself can alter the relationship between species richness and evenness [1], [42]. Nevertheless, we found a consistent negative relationship between species richness and evenness along the successional gradient. These results suggest that large-scale processes may dominate community structure in our successional meadows and thus generate the consistent pattern of the relationship between species richness and evenness. However it is important to note that our results are contrary to many investigations [7], [9], [12] which indicate that species richness is strongly positively correlated with evenness. As Stirling and Wilsey [1] pointed out, the discrepancy between the empirical explorations and the theoretical expectation may due to the lack of detailed empirical ecological processes in the latter [1]. They also suggested that the positive relationship between species richness and evenness in animal communities can switch to negative ones in plants and fungi [1]. In addition, successional changes can easily generate different patterns of the relationship between species richness and evenness.

### The Effect of Spatial Scale on the Relationship between Species Richness and Evenness

Our results clearly demonstrate that the strength of the relationship between species richness and evenness weakens with increase in spatial scale. Species richness and evenness are negatively corrected at relatively small scales (0.5 m×0.5 m to 2 m×2 m), but this negative relationship vanishes at larger scales (≥2 m×4 m). These results are in line with many previous investigations which indicate that scale can influence the measurement of species diversity and can therefore alter the observed relationship between species richness and evenness [21], [22], [43], [44], [45], [46]. However it’s important to note that, the negative relationship between species richness and species evenness at relatively small scales suggests that small scale disturbance such as selective foraging can have important effects on the structure of the plant communities of sub-alpine meadows.

### Species Evenness along the Successional Gradient

Our model fitting results indicate that among the models we tested, the niche-preemption model is the best predictor of the observed J′ along the successional gradient. Niche-preemption predicts that superior competitors tend to access greater amounts of limiting resources such as N and light and that other species adapt to the presence of a superior competitor by switching to an alternative, less-used resource [11], [47], [48], [49]. Many investigations have suggested that niche-preemption is the main mechanism of partitioning resources across the whole process of succession [50], [51], [52], [53], [54]. At the early stage of succession, due to a sparse canopy enough light is likely to be available, so light is not typically the limiting resource at early stages. Hence species with small seeds, rapid growth rate and short life-span are likely to invade and dominate communities, until other resources such as N, and moisture availability become limiting [55], [56], [57], [58]. As succession proceeds, the amount of light reaching the ground, available soil nutrients, and decomposition of branches and leaves all decline [50], [59], [60]. Abundant species in late successional communities are predicted to be superior competitors for these limiting resources [61]. Hence, abundant species in meadows of late successional stages are those with high SLA to maximize light capture [62]. Our model fitting results, together with the variation in species SLA along the successional gradient, suggest that niche-preemption induced dominating plasticity in resource use along the successional gradient may be the shaping mechanism of species evenness and its negative correlation with species richness. This mechanism may also explain the high diversity in sub-alpine meadows.

We argue that the relationships we found had to be inherently strong given that we obtained strong statistical significance despite the low power of our statistical tests. All relationships between species richness and evenness, and between SLA and species relative abundance along the successional gradient were explored in only 6 meadows of different successional ages, yet the differences in meadow successional age show clear consistent negative relationship between species richness and evenness (Fig. 1). Our results should therefore present strong evidence on the relationship between species richness and species evenness and also on the mechanisms of assembly of communities of high species diversity in the Qinghai-Tibetan Plateau.

Considered together, our results demonstrate that niche-preemption is an important mechanism that influences the pattern of the relationship between species evenness and richness along the successional gradient in sub-alpine meadows, and that this relationship is sensitive to the spatial scale of observation. However, stronger tests using field manipulative experiments and more detailed observations are needed to understand the mechanisms that determine species richness and community assembly in these alpine plant communities.

## Materials and Methods

### Ethics Statement

No permits were required to carry out this study.

### Study Site

A series of quadrat sampling were conducted in the sub-alpine meadow located in the eastern part of the Qinghai-Tibetan plateau, Hezuo, China (34°55**′**N, 102°53**′**E; 2900**m a.s.l.) in 2010. Mean annual precipitation of 530 mm is mainly distributed in the summer, and the mean annual temperature is 2.4°C. The vegetation at the study site is typical species-rich sub-alpine meadows, which is dominated by herbaceous species such as *Elymus nutans* Griseb, *Kobresia humilis* (C.A. Mey.) serg. and *Thermopsis lanceolate* R. Br [36]. Soils are classified as alpine meadow soils [36].

### Sampling Methods

Sampling was performed in year 2010 in one control meadow (no cropping) and three previously cropped meadows that had been abandoned 1, 3, 7, 9, and 10 years ago, with one meadow of each ‘age’ sampled. All meadows were located within an area of 4000 ha. We use the term ‘successional age’ to refer to the time since each meadow was protected from cropping. Meadows were selected so that meadows with similar successional ages were not closer to each other than to meadows with dissimilar ages. The area of each meadow was at least 120 ha, and had similar orientation, aspect and slope position. An area of 100 m×100 m plot was randomly selected in each meadow. Thirty 50 cm×50 cm quadrats were regularly arranged along the two transects, with 5 m intervals between adjacent quadrats. Grasses were sampled within quadrats in August during the peak of the growing season. In each quadrat, we recorded the number of ramets of each species and species richness.

One 10 m×10 m plot was installed in August during the peak of the growing season in 2011 in the control meadow. We divided each plot regularly into 100 squares of size 1 m×1 m using thin wires. Sampling was conducted at the scale of 0.05 m×0.05 m sub-quadrats. We collected the data of occurrence of species in the sub-quadrats to approximate each ramet of every species.

### Statistical Methods

#### Empirical test of the relationship between species richness and evenness

Here we calculate Pielou’s evenness J′ [63], which is expressed by the Shannon information scaled by the maximum information, to measure species evenness for each community:
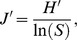
where H′ represents the observed value of Shannon index, and S is the total number of species observed. The reasons for choosing Pielou’s evenness J′ as the evenness index are twofold. First, it is the most widely used in ecology, and second, it is has been demonstrated in a study on a tropical forest that species richness predicted by species-abundance models increases with increasing evenness measured by J′ [7].The value of J′ ranges from 0 to 1, with larger values representing more even distributions in abundance among species. Jost [12] has shown that Pielou’s J′ is a good measure of ‘relative evenness’ and that relative evenness is the correct measure given the non-independence of richness and evenness. We use one-way ANOVA to test whether there exists significant variation in evenness (J′) along the successional gradient based on 30 samples in each meadow. Then we perform Spearman correlation analysis to explore the relationship between empirical S and J′ along the successional gradient.

#### Test of the effect of spatial scale on the relationship between species richness and evenness

To explore the effect of spatial scale on the relationship between species richness and evenness, we use Spearman correlation analysis to determine the relationship between S and J′ at different sampling scales in the 10 m×10 m plot. The data of species richness and evenness at a given spatial scale was sampled by continuously locating quadrats of different sizes on the 10 m×10 m plot in the control (natural meadow without farming) meadow. We selected 6 quadrat sizes which increase exponentially in area: 0.5 m×0.5 m (400 quadrats were sampled), 0.5 m×1 m (200), 1 m×1 m (100), 1 m×2 m (50), 2 m×2 m (25), 2 m×4 m (10).

#### The mechanism regulating species evenness in abundance along the successional gradient

For fitting species abundance models which have different evenness in abundance, we firstly created a set of communities ranging from the most even one where all the species share the same abundance, to the most uneven one in which the first species has abundance one less than the community size (*N−S*−1), and all other species (*S*−1) have only one ramet. Here *N* is the total number of ramets and *S* is the total number of species in the community. After that, for each successional meadow we set each of the 30 sampling quadrats (each of 0.5 m×0.5 m) as an objective community and then we use the species abundance data in the quadrat to estimate the parameters of four well-known species relative abundance models - the log-series, niche-preemption, TNBD (Truncated Negative Binomial Distribution), and broken-stick models (Table 3) and calculate the corresponding J′ of these six models for each quadrat in each meadow. Our species abundance data are from quadrats sampling, so this type of data is not entirely continuous. Hence here we use the bootstrap Kolmogorov-Smirnov test to compare the goodness-of-fit of the J′ values predicted by these models and the observed ones to test the mechanism of the observed J′ along the succession gradient theoretically. Also, analyses of the relationship between species-specific traits and relative abundance were conducted to test the occurrence of similar patterns for the best-fit model predictions and observed relationships between species traits and abundance along the successional gradients. This will help to determine whether the mechanisms invoked by the best-fit model can be applied to explain community assembly along the successional gradient. To carry out this test, we first log-transformed species’ relative abundances and specific leaf area to normalize the data and then we used Spearman correlation analysis to determine if there was a significant relationship between a species’ trait value and its relative abundance along the successional gradient. By these two analyses, we attempt to understand the processes that influence species evenness along the succession gradient. All above analysis are performed using package “biodiversity R” [64] and R ver. 2.12 (R Development Core Team).

## Supporting Information

Table S1 The result of the one-way ANOVA analysis of the variation of evenness along the successional age.
(DOCX)
