# Supplementary material for: The Relationship between Species Richness and Evenness in Plant Communities along a Successional Gradient: A Study from Sub-Alpine Meadows of the Eastern Qinghai-Tibetan Plateau, China
Source: PLoS One. 2012 Nov 9;7(11):e49024. doi: 10.1371/journal.pone.0049024 (PMC3494667; doi:10.1371/journal.pone.0049024)
Supplement: Table S1 — The result of the one-way ANOVA analysis of the variation of evenness along the successional age. (DOCX) [file pone.0049024.s001.docx]

Table S1

| Variance source | Degree of freedom | sum square | mean square | F-value | P-value |
| --- | --- | --- | --- | --- | --- |
| Successional age | 5 | 0.2644 | 0.0529 | 30.84 | 0.006 |
| Residuals | 174 | 0.2984 | 0.0017 |  |  |
| Total | 179 | 0.5628 |  |  |  |
